# Prevalence of HBV infection in immunocompromised patients on ART nearly equals HBV mono infection in individuals not infected with HIV, Shimla Northern India

**DOI:** 10.1186/1471-2334-14-S3-P26

**Published:** 2014-05-27

**Authors:** Aarti Garg, Vineeta Sharma, Santwana Verma, Digvijay Singh, Anil Kanga, Balraj Singh

**Affiliations:** 1 Indira Gandhi Medical College (IGMC), Shimla, Himachal Pradesh, India

## Background

It is estimated that 5% of the global population is chronically infected with HBV. The rate of HBV-HIV co infection is 10-20% in countries with HBV endemicity. The present study was undertaken to examine the HBV prevalence and HBV-HIV co infection at tertiary care institute, Northern India.

## Methods

This is a retrospective study for the duration of five years, 2008-13. The HBV infection was detected using HBsAg (Hepatitis B surface antigen) and HIV/AIDS cases were screened as per NACO, 2007 criteria.

## Results

A total of 10,134 cases were screened for detection of HBV infection and among these 1650(16.3%) were receiving ART (anti retroviral therapy) while HIV status was not divulged in 8484 (83.7%). HBsAg positive in 324/10134 (3.20%) cases, out of these HBV mono infection was seen in 264 (2.6%) and co- infection with HIV in 60 (0.6%).The concomitant HIV infections were seen in 3.64% (95 %confidence interval 2.8-4.7) while in HBV mono infections were in 3.1% (95 % confidence interval 2.8-3.4). There is no statistical difference among the two groups (*p* value >0.05). Among the pediatric age group 0.95% had concomitant HIV infections as against 5.2% with HBV infection alone. The mean age of presentation was 40.39 years. Males had higher prevalence rate of co infection (4.5:1).

## Conclusion

Nearly identical prevalence of HBV infection irrespective of HIV infection strongly indicates HIV screening for Hepatitis B infected patients. The presence of undetected co infection shall result in missed cases, incomplete or partial treatment and suboptimal clinical follow up.

